# Clinical observation of two bone cement distribution modes after percutaneous vertebroplasty for osteoporotic vertebral compression fractures

**DOI:** 10.1186/s12891-021-04480-6

**Published:** 2021-06-24

**Authors:** Qiujiang Li, Xingxia Long, Yinbin Wang, Tao Guan, Xiaomin Fang, Donggeng Guo, Jinhan Lv, Xuehua Hu, Xiaocheng Jiang, Lijun Cai

**Affiliations:** 1 Graduate School of Ningxia Medical University, Yinchuan, Ningxia China; 2 Department of Orthopedics, People’s Hospital of Ningxia Hui Autonomous Region, Lijun Cai, No. 56, Zhengyuan Street, Ningxia 750002 Yinchuan, China; 3 West China Hospital, Sichuan University, Sichuan, China

**Keywords:** Osteoporotic vertebral compression fractures, OVCFs, Percutaneous vertebralplasty, PVP, Bone cement distribution, Adjacent vertebral fracture, Vertebral body height

## Abstract

**Background:**

Current findings suggest that percutaneous vertebroplasty(PVP) is a suitable therapeutic approach for osteoporotic vertebral compression fractures (OVCFs). The present retrospective study aimed to investigate the differences in clinical efficacy and related complications between the two bone cement distribution modes.

**Methods:**

We retrospectively reviewed the medical records of the patients with single-segment OVCFs who underwent bilateral percutaneous vertebroplasty. Patients were divided into blocky and spongy group according to the type of postoperative bone cement distribution. Clinical efficacy and related complications was compared between the two bone cement distribution modes on 24 h after the operation and last follow-up.

**Results:**

A total of 329 patients with an average follow up time of 17.54 months were included. The blocky group included 131 patients, 109 females(83.2 %) and 22 males(16.8 %) with a median age of 72.69 ± 7.76 years, while the Spongy group was made up of 198 patients, 38 females(19.2 %) and 160 males(80.8 %) with a median age of 71.11 ± 7.36 years. The VAS and ODI after operation improved significantly in both two groups. The VAS and ODI in the spongy group was significantly lower than that in the blocky group, 24 h postoperatively, and at the last follow-up. There were 42 cases (12.8 %) of adjacent vertebral fractures, 26 cases (19.8 %) in the blocky group and 16 cases (8.1 %) in the spongy group. There were 57 cases (17.3 %) of bone cement leakage, 18 cases (13.7 %) in blocky group and 39 cases (19.7 %) in the spongy group. At 24 h postoperatively and at the last follow-up, local kyphosis and anterior vertebral height were significantly corrected in both groups, but gradually decreased over time, and the degree of correction was significantly higher in the spongy group than in the block group. The change of local kyphosis and loss of vertebral body height were also less severe in the spongy group at the last follow-up.

**Conclusions:**

Compared with blocky group, spongy group can better maintain the height of the vertebral body, correct local kyphosis, reduce the risk of the vertebral body recompression, long-term pain and restore functions.

## Introduction

With the aging of the social population,incidence of osteoporosis is constantly increasing,seriously affecting life quality of elderly patients [1]. Osteoporotic Vertebral Compression Fractures (OVCFs), one of the most common complications of osteoporosis, often occur in low energy damage or the absence of a clear trauma history,which primarily results in persistent back pain,local vertebral kyphosis,a reduced quality of life as well as increased mortality [2, 3]. The prevalence increases with age, with an estimated 140 000 new vertebral fractures per year in the United States [4, 5]. Percutaneous vertebroplasty can provide instant pain relief and stabilize the fractured vertebral body through the minimally invasive injection of polymethylmethacrylate(PMMA) bone cement, which has been widely used in OVCFs treatment [6, 7]. However, the risks related with vertebroplasty are not uncommon such as kyphosis, loss of height and adjacent segment fracture,and may be related to the type of bone cement distribution [6–9]. However, few studies have reported the relationship between bone cement distribution and imaging changes and clinical outcomes following PVP. Therefore, the present study aimed to investigate the differences in vertebral height correction, local vertebral kyphosis correction pain relief, functional recovery, etc. between the two bone cement distribution modes.

## Methods

### General data

The clinical data of patients with single-segment OVCFs who underwent bilateral percutaneous vertebroplasty from April 2016 to April 2019 were retrospectively reviewed. The inclusion criterias are as follows: 1.the patient had obvious back pain, and limited physical activity, especially in cases of turning over or getting up. 2.T score ≤ − 2.5 at spine/hip at Dual energy X-ray absorptiometry (DXA). 3.The signal change of the lumbar fracture by lumbar magnetic resonance imaging(MRI) suggesting a hyperintense T2 signal and a hypointense T1 signal,or a whole-body bone scan performed a active bone metabolism. Exclusion criterias are as follows: 1.Patients with OVCFs caused by tumor, infection, or tuberculosis. 2.Patients had coagulation dysfunction,combined systemic disease,and inability to tolerate the procedure. 3. Systemic or local infection. 4. Spinal cord compression and obvious neural symptoms such as numbness and/or muscle weakness. 5. Incomplete follow-up data. We eventually recruited 392 patients, including 60 males and 269 females, mean age 68.21 ± 10.48 years, and mean follow-up time 17.54 months. The present study was approved by the medical ethics committee of the Peoples Hospital of Ningxia Hui Autonomous Region. All included patients signed an informed consent.

### Surgical method

The patient was placed in the prone position, the abdomen was vacated, and the fractured vertebrae were located under C-arm fluoroscopic guidance. The puncture needle was inserted into the vertebral body via bilateral arch pathways. The tip of the puncture needle was located in the anterior middle third of the vertebral body on the lateral view, and the anterior-posterior view was located between the inner edge of the ipsilateral pedicle and the vertebral body midline. The working channel is established and the high-viscosity cement is slowly injected under C-arm fluoroscopy until the bone cement approaches the posterior wall of the vertebral body where leakage may occur, and the working channel is slowly withdrawn after the cement has hardened. The whole procedure was done with the assistance of C-arm fluoroscopy. All patients were given oral calcium and vitamin D postoperatively and an intravenous infusion of Zoledronic acid (Aclasta, 100ml/5 mg) once a year thereafter for 3 years. Patients were reviewed on the postoperative 24 h for anteroposterior and lateral radiographs and discharged 2 to 3 days after surgery. X-ray films of injured vertebra were reviewed periodically after surgery.

### Grouping method

**Fig. 1:**
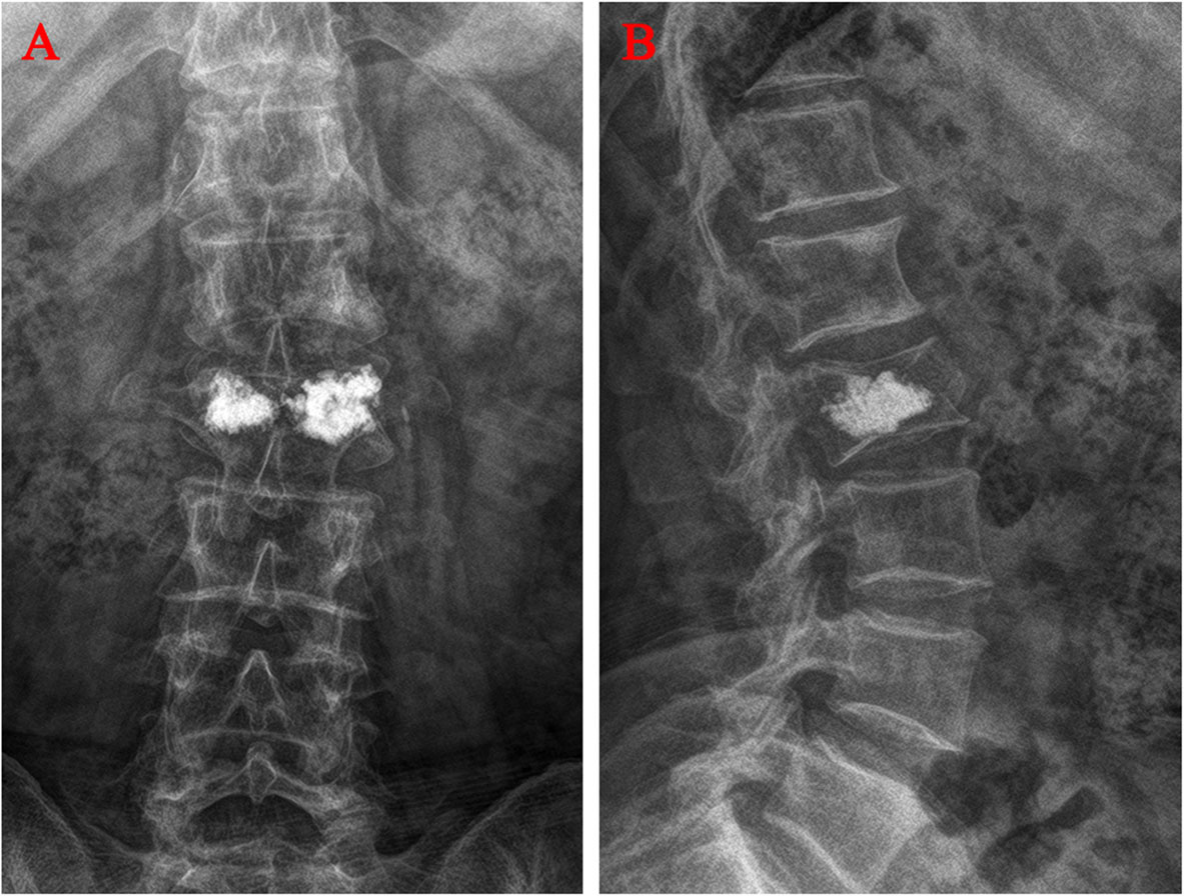
Distribution characteristics of blocky bone cement. **A** Anteroposterior X-ray film of local solid distribution pattern in the blocky group. **B** Lateral X-ray film of local solid distribution pattern in the blocky group

**Fig. 2:**
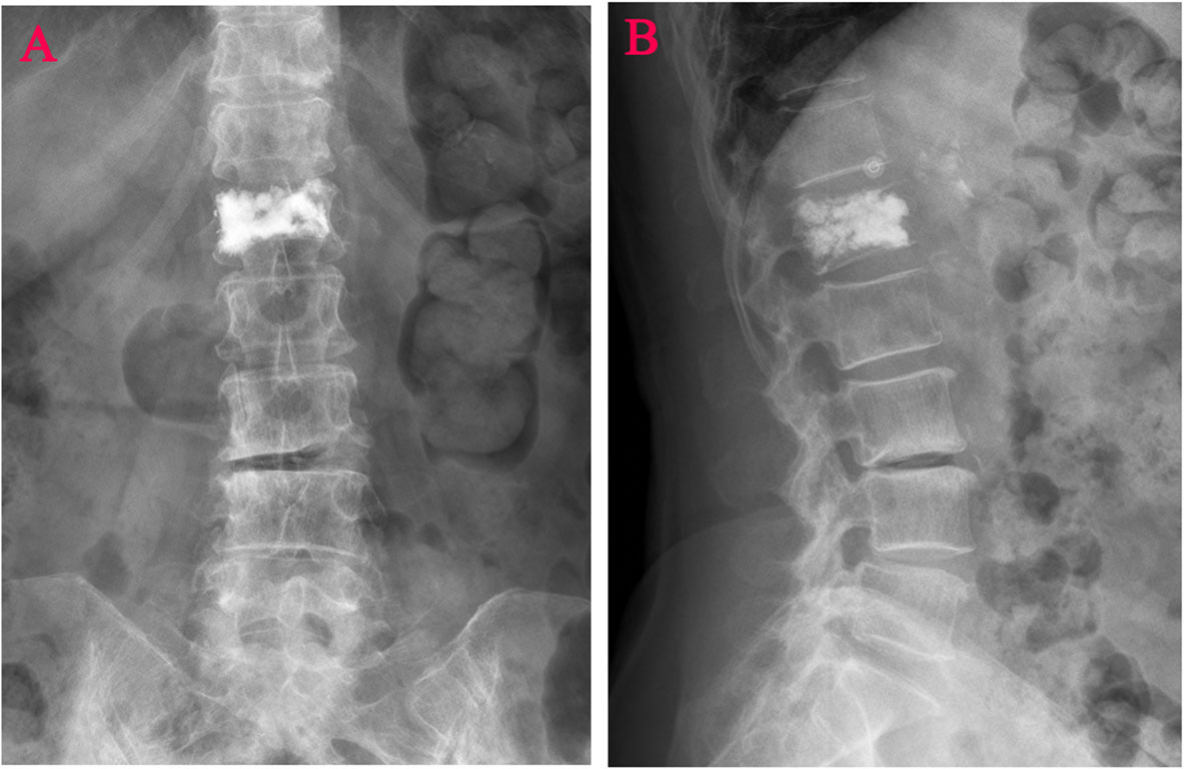
Distribution characteristics of spongy bone cement. **A** Anteroposterior X-ray film of diffuse distribution pattern in the spongy group. **B** Lateral X-ray film of diffuse distribution pattern in the spongy group

Anteroposterior and lateral radiographs were taken 24 h after surgery and divided into blocky group (Fig. 1) and spongy groups (Fig. 2) according to the difference in the distribution of bone cement in the vertebral body on the X-ray images after PVP treatment [10].The vertebrae in which bone cement localized compact and solid distribution in anteroposterior and lateral X-ray film comprised the blocky group (Fig. 1). The vertebrae in which bone cement localized diffuse, fibril-like and sponge-like distribution in anteroposterior and lateral X-ray film comprised the spongy group (Fig. 2).

### Evaluation method

**Fig. 3:**
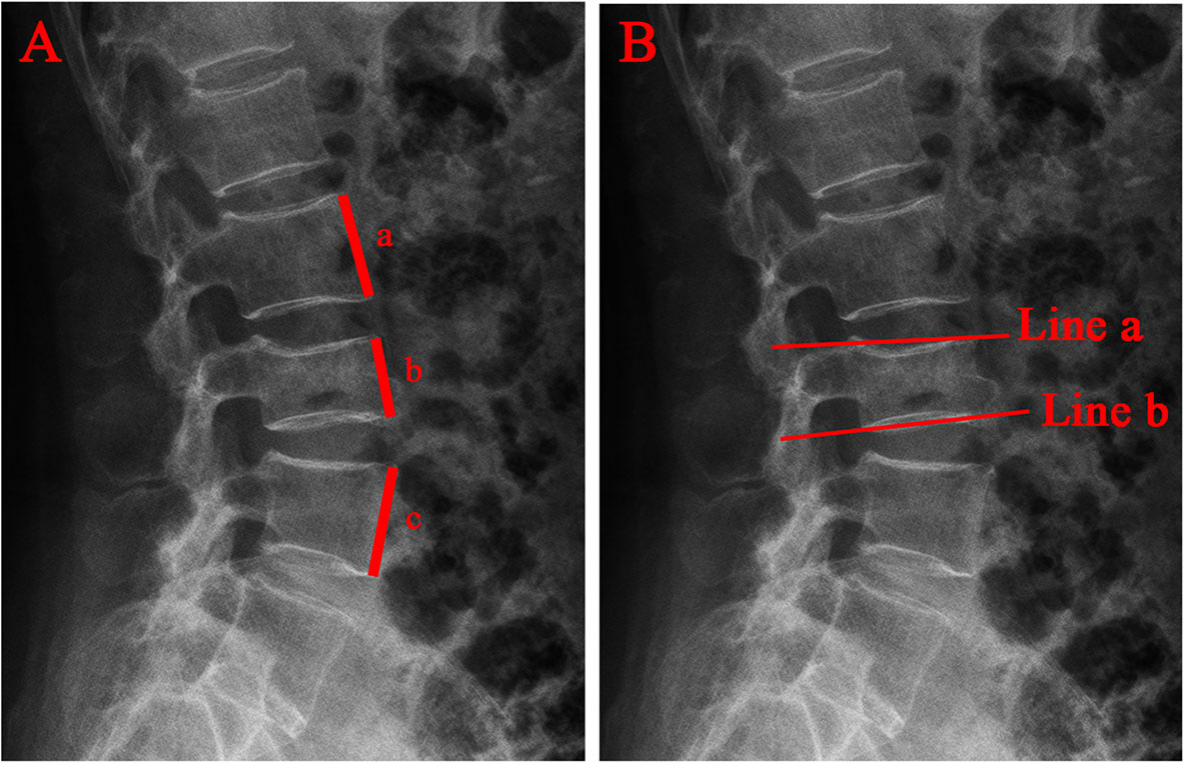
Radiographic evaluation of compressed vertebrae. **A** The anterior vertebral height ratio(AVHR) was defined as the height of the anterior wall of the compressed vertebral body(b) / (the height of the anterior wall of the upper vertebral body(a) + the height of the anterior wall of the lower vertebral body(c))×2. **B** Cobb angle was defined as the angle formed by the upper endplates(Line a) and lower endplates(Line b) of the fractured vertebral body

Gender, nationality, age, diabetes history, hypertension history, fracture history, body mass index (BMI), Bone mineral density(BMD), fracture segment, follow up time, bone cement volume, operation duration, blood loss, adjacent vertebral fracture and bone cement leakage were documented. The Oswestry Disability Index (ODI) and Visual Analog Scale (VAS) were recorded to assess the clinical outcomes before surgery, 24 h after surgery and at the last follow-up. The anterior vertebral height (AVH) and local kyphotic angle (LKA, Cobb’s method) of the fractured vertebral body were measured before surgery, 24 h after surgery and at the last follow-up. AVH change was defined as postoperative AVH - preoperative AVH. The anterior vertebral height ratio(AVHR) was defined as the height of the anterior wall of the compressed vertebral body / (the height of the anterior wall of the upper vertebral body + the height of the anterior wall of the lower vertebral body)×2 (Fig. 3). The anterior vertebral height recovery ratio(AVHRR) was defined as postoperative AVHR - preoperative AVHR. The anterior vertebral height loss ratio(AVHLR) was defined as postoperative AVHR-last follow-up AVHR. Cobb angle was defined as the angle formed by the upper and lower endplates of the fractured vertebral body (Fig. 3). Local kyphotic angle change was defined as last follow-up Cobb angle – postoperative Cobb angle. To determine the intraobserver and the interobserver reliability of the measurements, two of the authors(XC J and XH H) performed blinded measurements. The average values of the measurements were used for analysis.

### Statistical analysis

Inter- and intraobserver reliabilities for all measure-ments were calculated using the intraclass correlation coefficient(ICC) and the ICC > 0.75 indicates good reliability [11]. The data were statistically analyzed using SPSS 22.0 software (SPSS, Inc., USA). Categorical variables were expressed as rates, and the chi-square test was used for comparison between groups. Continuous variables were expressed as mean + standard deviation, and independent samples t-test or analysis of variance (ANOVA) was used for comparison between groups. Differences were defined as statistically significant at *P* < 0.05.

## Results

The ICCs for inter- and intraobserver reliability were > 0.90 for all measurements, which was defined to be an excellent reliability.

A total of 329 patients with an average follow up time of 17.54 months (range from 11 to 36 months) were included. The blocky group included 131 patients, 109 females(83.2 %) and 22 males(16.8 %) with a median age of 72.69 ± 7.76 years, while the Spongy group was made up of 198 patients, 38 females(19.2 %) and 160 males(80.8 %) with a median age of 71.11 ± 7.36 years. There were 28 cases (8.51 %) of intravertebral cleft(IVC), 16 cases (12.2 %) in blocky group and 12 cases (6.1 %) in spongy group (Table 1). The incidence of IVC in blocky group was higher than that in spongy group (Table 1). There was no significant difference in gender, nationality, age, comorbidities(diabetes history, hypertension history, fracture history), BMI, BMD, fracture segment, follow up time, bone cement volume, operation duration and blood loss, as depicted in Table 1.

**Table 1:** Comparison of baseline characteristics of patients between two groups

	Blocky group	Spongy group	t/χ^2^-value	*P*-value
**Gender**			0.304	0.581
Male	22(16.8 %)	38(19.2 %)		
Female	109(83.2 %)	160(80.8 %)		
**Nationality**			0.117	0.732
Han	118(90.1 %)	176(88.9 %)		
Hui	13(9.9 %)	22(11.1 %)		
**Age(years)**			3.968	0.265
<60	4(3.1 %)	12(6.1 %)		
60 ~ 70	47(35.9 %)	71(35.9 %)		
70 ~ 80	54(41.2 %)	89(44.9 %)		
>80	26(19.8 %)	26(13.1 %)		
**Diabetes history(%)**	9.2 %	8.6 %	0.032	0.857
No(cases)	119	181		
Yes(cases)	1	17		
**Hypertension history(%)**	48.9 %	44.9 %	0.483	0.487
No(cases)	67	109		
Yes(cases)	64	89		
**Fracture history(%)**	16.8 %	15.7 %	0.075	0.784
No(cases)	109	167		
Yes(cases)	22	31		
**BMI(kg/m**^**2**^**)**	24.25 ± 3.61	23.65 ± 3.42	1.537	0.125
**Bone mineral density (T score)**	-3.23 ± 0.85	-3.26 ± 0.90	0.260	0.795
**Fracture segment**			3.544	0.170
Thoracic (T1 ~ 9)	10(7.6 %)	20(10.1 %)		
Thoracolumbar (T10 ~ L2)	78(59.5 %)	97(49.0 %)		
Lumbar (L3 ~ 5)	43(32.8 %)	81(40.9 %)		
**Intravertebral cleft(%)**	12.2 %	6.1 %	3.834	0.050
No(cases)	115	186		
Yes(cases)	16	12		
**Follow up time(months)**	17.83 ± 7.33	17.35 ± 6.54	0.618	0.537
**Bone cement volume(mL)**	4.15 ± 1.16	4.11 ± 1.11	0.264	0.792
**Operation duration(mins)**	42.6 ± 12.04	41.73 ± 11.12	0.67	0.503
**Blood loss(mL)**	11.17 ± 3.66	11.07 ± 3.58	0.239	0.811

There were no statistically significant differences in the VAS and ODI between the two groups before the operation,but a significant reduction in pain was reported in the blocky and spongy group after surgery (Table 2).The VAS and ODI in the spongy group was significantly lower than that in the blocky group, 24 h postoperatively, and at the last follow-up (Table 2).

**Table 2:** Analysis of outcome between two groups

	Blocky group	Spongy group	T-value	*P*-value
**VAS**				
pre-op	6.29 ± 0.90	6.28 ± 1.05	0.060	0.948
24 h post-op	2.54 ± 0.91	2.12 ± 0.79	4.355	< 0.001
last follow-up	2.13 ± 0.738	1.81 ± 0.67	4.090	< 0.001
**ODI**				
pre-op	63.63 ± 11.94	61.02 ± 11.79	1.958	0.051
24 h post-op	35.88 ± 6.72	33.84 ± 6.38	2.773	0.006
last follow-up	26.78 ± 5.53	23.2 ± 7.59	4.941	< 0.001
**AVH(mm)**				
pre-op	14.60 ± 2.96	15.22 ± 3.16	-1.785	0.075
24 h post-op	16.72 ± 3.12	17.62 ± 3.19	-2.505	0.013
last follow-up	16.23 ± 3.13	17.14 ± 3.25	-2.545	0.011
**Cobb(%)**				
pre-op	26.71 ± 6.91	27.08 ± 7.55	-0.465	0.642
24 h post-op	17.94 ± 3.51	15.85 ± 4.03	4.847	< 0.001
last follow-up	24.31 ± 4.92	21.04 ± 4.59	6.142	< 0.001
**AVHR(%)**				
24 h post-op	54.95 ± 10.61	58.17 ± 10.55	-2.703	0.007
last follow-up	46.58 ± 10.96	50.55 ± 10.84	-3.237	0.001
**AVH change(mm)**				
24 h post-op	2.13 ± 0.73	2.40 ± 0.69	-3.427	0.001
last follow-up	1.63 ± 1.078	1.93 ± 1.08	-2.466	0.014
**AVHLR(%)**	8.37 ± 2.97	7.62 ± 2.96	2.245	0.025
**AVHRR(%)**	6.99 ± 2.42	7.94 ± 2.35	-3.544	< 0.001
**Local kyphotic angle change(°)**	6.37 ± 4.27	5.20 ± 3.81	2.553	0.011
**Adjacent vertebral fractures(%)**	19.8 %	8.1 %	9.802	0.002
No (cases)	105	182		
Yes (cases)	26	16		
**Bone cement leakage (%)**	13.7 %	19.7 %	1.953	0.162
No (cases)	113	151		
Yes (cases)	18	39		

There were 42 cases (12.8 %) of adjacent vertebral fractures, 26 cases (19.8 %) in blocky group and 16 cases (8.1 %) in spongy group. The incidence of postoperative adjacent vertebral fractures in spongy group was significantly lower than that in blocky group, and the difference was statistically significant (Table 2). There were 57 cases (17.3 %) of bone cement leakage, 18 cases (13.7 %) in blocky group and 39 cases (19.7 %) in spongy group (Table 2). There were no clinical symptoms between two groups, and the difference was not statistically significant.

Local kyphotic angle change was significant smaller in spongy group (Table 2). AVHHR in spongy group is higher than that in blocky group, it was significant difference in AVHRR between the two groups (Table 2). At the last follow-up, AVHLR in spongy group was significantly lower than that in blocky group. AVH and AVHR were all significantly restored at 24 h and last follow-up after surgery, compared with the preoperative data in groups (Table 2). Similarly, Cobb angle improved significantly after surgery for both groups (Table 2). At the 24 h and last follow-up, AVH, AVHR,AVH change in spongy group were significantly higher than that in blocky group, while Cobb angle was significantly lower (Table 2).

## Discussion

In 1987, Galibert first used vertebroplasty to treat C2 vertebral hemangioma [12]. Studies have been reported on PVP for OVCFs in 1988 [13] and 1994 [14], respectively. Since then, the technique has been developed and refined, and it has gradually become the main methods for treating OVCFs due to its simplicity and efficacy. PVP strengthens the vertebral body by injecting bone cement into the fractured vertebral body to restore the height, strength, and stiffness of the vertebral body, while correct the local kyphosis and produce a thermal effect on the nociceptive nerves around the vertebral body to rapidly relieve pain symptoms [15, 16]. However, this method is affected by many factors, such as BMD, the amount, distribution, and leakage of bone cement, etc [17].

A biomechanical study of 120 vertebrae from 10 osteoporotic female cadavers found, on average 16.2 and 29.8 % of the vertebral cement filling is required to restore strength and stiffness, respectively, and it was no correlation between the recovery of vertebral strength and stiffness and the percentage volume of bone cement filling [18]. Liebschner [19] et al. performed a single lumbar PVP finite element analysis study, which found that only a small amount of bone cement (14 % volume) was required to restore the stiffness of the fractured vertebrae to pre-injury levels, and that a larger volume of bone cement did not provide greater benefit, as the increase in vertebral strength with bone cement resulted in asymmetric distribution of bone cement and strength imbalance on both sides of the vertebral body. Related studies also confirm the above-mentioned view [20, 21]. In addition, the correlation between bone cement volume and surgical outcome is small, and an increase in cement volume may increase the risk of cement leakage [22]. Bone cement injection volume is a one-sided indicator of the benefit of bone cement and does not reflect the distribution of bone cement within the vertebral body. Therefore, it is important to study the distribution of bone cement and the clinical outcome and prognosis of PVP. However, few studies have reported the effect of bone cement distribution on radiographic and functional recovery after PVP treatment.

Compared to PVP, PKP results in poorer cement distribution and a greater likelihood of postoperative vertebral height loss [23]. The main reason is that the expansion of the balloon compresses the more lax cancellous bone in the cone, thus creating a “cavity” at the balloon site, and the injected bone cement tends to be distributed in this low-pressure cavity without dispersing into the surrounding bone, making it difficult to bind tightly to the cancellous bone. Therefore, this blocky distribution of bone cement has been shown to be an important factor in vertebral body height loss. The subjects selected in this study were all post-PVP patients, excluding the influence of the surgical approach on the results.

A study by Chen et al. [24] reported that the symmetric distribution of bone cement is closely related to the stiffness of the vertebral body, and in unilateral vertebroplasty, the bone cement is often confined to the ipsilateral side of the vertebral body and cannot effectively diffuse across the midline; therefore, the end result can be a significant reduction in the stiffness of the vertebral body on the unreinforced side of the bone cement compared to the reinforced side. The biomechanical imbalance can exacerbate the pressure load on the spine, resulting in effects that are difficult to reverse, such as loss of height of the fractured vertebral body, disc degeneration in adjacent segments, and even fracture of the vertebral body in adjacent segments [25, 26]. Therefore, all subjects included in this study received bilateral arch root PVP surgery, further reducing the detrimental effects of asymmetric distribution of bone cement in the coronal plane. The majority of females in this study indicated that postmenopausal women are more likely to have osteoporosis in patients. The majority of the study population(81.76 %, 269/329) was comprised of females, suggesting that postmenopausal women are more likely to suffer from osteoporosis.

Spinal imaging changes such as vertebral body height and Cobb angle are often used as indicators to assess the efficacy of PVP [27]. Yan et al. [28] reported that PVP was able to restore vertebral body height and correct kyphosis. The results of the present study showed that PVP was able to significantly restore anterior vertebral body height and reduce the Cobb angle without considering the cement distribution, and if the cement was spongy in the vertebral body, it could better maintain the height of the vertebral body and reduce the risk of postoperative vertebral body height loss as well as local kyphosis. There are numerous factors that contribute to enhanced postoperative vertebral body height loss and increased kyphosis that do not require exposure to a traumatic event and may be related to the severity of osteoporosis, daily activity level, and cement distribution [25]. However, bone cement distribution is an important factor contributing to vertebral height loss [9]. He et al. [23] reported that the incidence of vertebral height loss was higher in the uninterlocked solid pattern of bone cement distribution than in the interlocked solid pattern. Furthermore, Yu et al.  [8] also confirmed that the comparatively diffused pattern of bone cement distribution has better medium and long-term clinical outcomes, compared to the solid lump distribution pattern. The spongy bone cement distribution allows cancellous bone and bone cement to more fully interlock and increases vertebral strength and stiffness with greater homogenization, thus reducing the risk of vertebral height loss after PVP [29]. Our study also found a loss of vertebral body height and local kyphosis over time in the blocky and spongy group postoperatively, which is consistent with previous findings [30, 31]. In contrast, the blocky group showed more pronounced changes on imaging than the spongy group, which was related to the distribution of bone cement.

Our study is consistent with previous studies reporting [32, 33] that PVP significantly relieves short-term pain and restores function, regardless of the bone cement distribution pattern. In long-term follow-up, it was also found that VAS and ODI scores were significantly lower in the spongy group than in the blocky group, suggesting that spongy bone cement distribution has better analgesic and functional recovery effects. The cause of persistent lower back pain is mainly related to insufficient filling of the bone cement [34]. The bone cement does not bind effectively to the fractured vertebrae, and the low strength and stiffness of the vertebrae are not sufficient to provide effective support, resulting in a continuous loss of height [35, 36]. Therefore, we speculate that the spongy distribution of the bone cement allows greater contact with the cancellous bone within the vertebral body, which can adequately immobilize the fractured fragment, increasing spinal stability and reducing micromovement of the trabeculae, thus reducing pain and achieving functional recovery [34, 37].

Liebschner [19] et al. reported that the recovery of vertebral body strength was closely related to the distribution of bone cement. The strength and stiffness of the vertebral body after bone cement strengthening are significantly higher than the adjacent vertebrae, and the inhomogeneous distribution of bone cement makes the strength and stiffness of the vertebral body asymmetrical in all areas, and all of these factors tend to increase the risk of fracture of the adjacent vertebral body after PVP [24, 38, 39]. Therefore, numerous studies have confirmed that homogeneous distribution of bone cement within cancellous bone can reduce stress concentration and thus reduce the risk of fracture in adjacent vertebrae [29, 40]. The spongy group has a spongy and homogeneous distribution of bone cement, which can fill the cancellous bone better and reduce the concentrated stress between adjacent vertebrae. Our results confirmed that the incidence of adjacent vertebral fractures was significantly lower in the blocky group than spongy. Therefore, achieving good distribution of bone cement within cancellous bone is crucial in reducing the risk of adjacent vertebral fractures.

There is an association between the distribution of bone cement and cement leakage [29]. The overall bone cement leakage rate in our findings was consistent with the results reported in previous studies [41], and most patients were clinically asymptomatic. The rate of bone cement leakage was lower in the blocky group than spongy, but the difference was not statistically significant. This may be the difference of the leakage sites.The spongy cement distribution is more widely distributed than the blocky group and more likely to leak through the broken bone cortex or endplate to the intervertebral disc or paravertebral area, while the incidence of leakage in the anterior edge of the vertebral body of the blocky group was the highest [40]. Therefore, the surgeon should carefully analyze the imaging data preoperatively and should suspend the procedure in case of intraoperative cement leakage.

### Limitations

This study currently has some limitations. First, our study is a retrospective study with a relatively small sample size, which may result in some bias. Second, the grouping method in our study differs from previous studies, in which it may lead to subjective bias in the results. Therefore, multicenter, prospective studies with larger samples are needed to further elucidate the relationship between intravertebral bone cement distribution and clinical outcomes of PVP.

## Conclusion

Both groups of bone cement distribution have good immediate analgesic effect. However, compared with the blocky group, the spongy group could better maintain the height of the vertebral body, correct local kyphotic, improve function, reduce the risk of postoperative adjacent vertebral fractures, and it was more effective.

## Data Availability

Please contact the corresponding author for data requests.

## Abbreviations

OVCFs: Osteoporotic Vertebral Compression Fractures;
PVP: Percutaneous vertebroplasty
PMMA: Polymethylmethacrylate
DXA: Dual energy X-ray absorptiometry
MRI: Magnetic resonance imaging
BMD: Bone mineral density
BMI: Body mass index
ODI: Oswestry Disability Index
VAS: Visual Analog Scale
AVH: Anterior vertebral height
LKA: Local kyphotic angle
AVHR: Anterior vertebral height ratio
AVHRR: Anterior vertebral height recovery ratio
AVHLR: Anterior vertebral height loss ratio
